# A Simple Replica Method as the Way to Obtain a Morphologically and Mechanically Bone-like Iron-Based Biodegradable Material

**DOI:** 10.3390/ma15134552

**Published:** 2022-06-28

**Authors:** Marlena Grodzicka, Gabriela Gąsior, Marek Wiśniewski, Michał Bartmański, Aleksandra Radtke

**Affiliations:** 1 Faculty of Chemistry, Nicolaus Copernicus University in Toruń, Gagarina Street 7, 87-100 Toruń, Poland; marlena.grodzicka@yahoo.com (M.G.); ggasior.szop@gmail.com (G.G.); marekw@umk.pl (M.W.); 2 Faculty of Mechanical Engineering and Ship Technology, Gdańsk University of Technology, Narutowicza Street 11/12, 80-233 Gdańsk, Poland; michal.bartmanski@pg.edu.pl

**Keywords:** iron, biodegradable implants, surface energy, albumin adsorption

## Abstract

Porous iron-based scaffolds were prepared by the simple replica method using polyurethane foam as a template and applying the sintering process in a tube furnace. Their surface morphology was characterized using scanning electron microscopy (SEM) and phase homogeneity was confirmed using X-ray diffraction (XRD). Corrosion behavior was determined using immersion and potentiodynamic polarization methods in phosphate buffered saline (PBS). The surface energy was calculated by studying the changes of enthalpy of calorimetric immersion. A preliminary biological test was also carried out and was done using the albumin adsorption procedure. Results of our work showed that in using the simple replica method it is possible to obtain iron biomaterial with morphology and mechanical properties almost identical to bones, and possessing adequate wettability, which gives the potential to use this material as biomaterial for scaffolds in orthopedics.

## 1. Introduction

Bone implants are commonly used not only for bone fractures caused by accidents, but also after the removal of bone fragments, e.g., facial bone affected by cancer [1]. Implants made of titanium alloys, stainless steel, and CoCrMo alloys are most commonly used [2,3]. It should be considered that orthopedic implants, which are implanted in the human body, are not implants that should be there permanently [4]. There are an increasing number of reports on the harmfulness of implants, which are present in the body for long periods of time [5].

Biodegradable or bioresorbable implants are the new trend in implantology. Iron-, zinc-, and magnesium-based materials have received a lot of attention [6,7,8]. A significant aspect in implantology is the concept of biocompatibility, understood as the absence of toxic or unfavorable immunological reactions when implants are in contact with cells or bodily fluids [9]. It is extremely probable that a material with bone-like properties will be a biocompatible material. It is important during the design of materials, which are going to be used in orthopedics to pay attention to the bone structure. The main component of bones is type I collagen (40% by volume) interspersed with mineral crystals consisting of non-stoichiometric calcium hydroxyapatite (45%). The remaining volume (15%) is occupied by water, which is bound to the collagen or located in spaces called the lacunocanalicular system [10]. Due to this structure, the bone has adequate mechanical properties and it is able to resist pressure and tension [11]. Materials that are bioresorbable and can be used in implantology are mainly polymers, bioceramics, and metals; however, due to the properties mentioned above, mainly mechanical ones, only metals can be considered as scaffolds in orthopedics [12]. Among metals, iron is especially investigated as a biodegradable scaffold, as it is highly resistant, ductile, and formable [13,14]. Biodegradable iron-based biomaterials should degrade in the human body at a suitable rate. It would be ideal, if the rate of degradation was similar to the rate of new bone formation. It would allow for a gradual transfer of loads from the implant to the new bone [15,16]. In bodily fluids the degradation of iron involves anodic and cathodic reactions, as shown in Equations (1) and (2):

Fe → Fe^2+^ + 2e^−^ (anodic reaction) (1)

O_2_ + 2H_2_O + 4e^−^ → 4OH^−^ (cathodic reaction) (2)

The ions Fe^2+^ and OH^−^ according to the Pourbaix diagram combine to form Fe(OH)_2_, which is the main corrosion product (Equation (3)) [17].

Fe^2+^ + 2OH^−^ → Fe(OH)_2_ (3)

Due to the presence of ions other than hydroxyl in bodily fluid, e.g., chlorides, phosphates, it is concluded that a number of reactions may take place to form compounds such as: Fe_3_(PO_4_)_2_ 8H_2_O, Fe(OH)_3_, Fe(PO_4_) [18]. The availability of oxygen in the tissue in the area around the implant is a crucial condition for degradation and a determining parameter for the rate of corrosion of the iron implant in vivo [19].

In the human body, iron has an important role in many physiological processes, including DNA metabolism, oxygen transport, and cellular energy production [20]. Due to the importance and presence of iron in the human body there are many proteins and mechanisms involved in the management of iron. The three most important are transferrin, which is responsible for the transport and recycling of iron; ferritin, which protects the penetration of iron into the body and keeps excess iron in a safe and bioavailable form; and hepcidin, which belongs to the proteins produced in the liver and plays a special role in regulating the entry of iron into circulation, regulates intestinal iron absorption, plasma iron concentration, and iron distribution in tissues [21,22,23]. In the case of a biodegradable material, it is crucial that the amount of iron released is managed by the organism in the way known from homeostasis and that the amount of ions released does not exceed the critical value for the cells [24].

The corrosion rate is affected not only by the chemical composition of the material but also by its morphology. Porous materials have a faster rate of degradation than non-porous materials such as roll-formed plates. This is an effect of the larger surface in contact with the physiological environment and thus more locations where corrosion can be initiated. High porosity can also have an effect by increasing crevice corrosion [25]. There are quite a few variations in methods to obtain porous composites, such as 3D printing, electroforming, or the repeated template method [26,27]. For 3D printing, there is a necessity for a computer project design (for example, in a CAD program) to specify the material size, pore diameter, and other parameters of the target material. There are several different publications with results of successful 3D printing used to obtain porous iron-based biodegradable materials. Samples prepared in this way are characterized by high accuracy and consistency with the designed pattern. Limitations of the method are using an expensive printer and the availability of materials [28,29,30]. Electroforming is a method where metal is dissolved electrolytically at the anode, and metal ions are transported by an electrolyte solution to the cathode. In this way, there is a possibility to position a metal on or against a mandrel. This method is very similar to electroplating; however, electroplating is about taking an existing device and applying a metallic layer (coating), and electroforming’s main goal is to create a new object [31]. There are reports of using this method to obtain materials from iron and other metals designed as implants [32,33,34]. Electroforming seems to be promising in the future perspective of biodegradable metal implants. The repeated template method is a method that is very simple to carry out and modify. It consists of applying metal powder on the (usually polymer) template and then heating it in a furnace until the metal particles melt together and destroy the template. Metal foams can be obtained by using a template with a porous structure [35,36]. The main advantage of this process is its ease and the possibility of obtaining samples with a large variation in porosity, but at the same time, the full dependence on the template for the shape and the inability to modify the shape of foam afterwards can be seen as a disadvantage of this method [37]. We decided to work with the last method and check the similarity of the material obtained with bone. In this paper we would like to present the first part of our study on iron-based biodegradable material obtained in a very simple replica method—its preparation and characterization in terms of the morphology, surface energy, mechanical properties, chemical and electrochemical degradation, as well as a primary biological test using the albumin adsorption procedure. The second part of our research involving the biological test will be the topic of our next paper.

## 2. Materials and Methods

### 2.1. Fe Scaffolds Preparation

**Figure 1:**
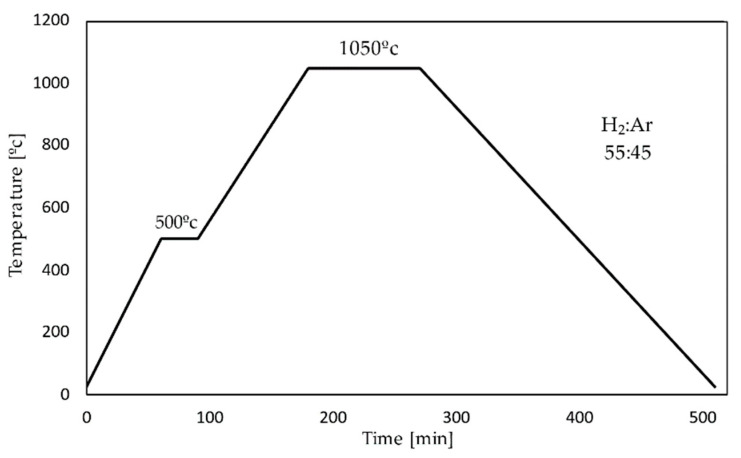
Scheme of the used heat treatment—temperature program; heating rate—step I—7.9 °C/min, step II—6.1 °C/min; cooling rate—4.3 °C/min.

The materials studied in this work were macro-porous Fe scaffolds. They were made of iron powders with particle sizes <10 micron (purity 99.9%, Alfa Aesar, Kandel, Germany). The powders were mixed with a solution of 5% polyvinyl alcohol (M_w_ 89,000–98,000, 99+% hydrolyzed, Sigma Aldrich, Darmstadt, Germany) in a mass ratio of 1:2. Then the templates made of polyurethane PU PPI45 (sheet of polyurethane PU PPI45 filter foam, size 2000 mm × 1000 mm × 5 mm with a density of 45 channels per inch, where 97% of the pores are open pores, cut into cubes 6 mm × 6 mm × 6 mm; Rekuperator, Wejherowo Poland) were soaked and squeezed out of a porous polyurethane sponge. They were put in a laboratory dryer and dried for 30 min at 50 °C. Then they were soaked in the mixture again. The systems prepared in this way were placed in a tube furnace (Czylok, Jastrzębie-Zdrój, Poland) and sintered for 5 h according to the temperature program shown in Figure 1. All samples were ultrasonically cleaned in acetone (ACS reagent, ≥99.5%, Sigma Aldrich, Darmstadt, Germany) and ethanol (anhydrous, ≥99.5%, Sigma Aldrich, Darmstadt, Germany) for 10 min each, followed by argon drying before being subjected to testing. The prepared samples were stored in a desiccator until use.

### 2.2. Fe Scaffolds Characterization

The morphology of the produced scaffolds was studied using a Quanta scanning electron microscope with field emission (SEM, Quanta 3D FEG, Huston, TX, USA). Phase homogeneity was confirmed using X-ray diffraction (XRD, Philips X “Pert with X’Celerator Scientific detector) using CuKα radiation and an incident angle of 2◦. Elemental analysis was illustrated using energy-dispersive X-ray spectroscopy (Quantax 200 XFlash 4010, Bruker AXS, Karlsruhe, Germany)). Low temperature N_2_ adsorption isotherms were measured using an ASAP2010 volumetric adsorption analyzer from Micromeritics (Nor-cross, GA) at liquid nitrogen temperature (77 K) in the relative pressure range from about 10^−6^ up to 0.999. Before the measurements, the samples were outgassed for 2 h at a temperature of 373 K.

### 2.3. Immersion Enthalpy and Surface Energy Determination

The measurements were performed using a Tian–Calvet isothermal calorimeter constructed in our laboratory [38]. N-heptane, deionized water, and formamide were used as standards. Each measurement was repeated at least three times. The major assumption of the van Oss-Good-Chaudhury (VGC) model used in this study is the independency of the dispersive and acid-base interactions. Equation (4) describes the enthalpy of immersion to the surface energy components of both the solid surface and the wetting liquid.

(4) $$-h_{i}=-H_{l}+2\left[\sqrt{H_{S}^{LW}\cdot H_{l}^{LW}}+\sqrt{H_{S}^{+}H_{l}^{-}}+\sqrt{H_{S}^{-}H_{l}^{+}}\right]$$

where *h_i_* represents the enthalpy change upon immersion (mJ/m^2^); *H* is the surface enthalpy (mJ/m^2^); subscripts *S, l* are the solid surface and wetting liquid respectively; superscripts *LW*, +, − are the Lifshitz-Van der Waals, acidic and basic components of surface energy, respectively.

Moreover, the second component of the sum in (4) represents the work of the particular liquid adhesion, thus one can write:

(5) $$-h_{i}=-H_{l}+W_{adh}$$

The energy components of the different probe liquids used in this study were taken from [39].

### 2.4. Nanomechanical Properties of Fe Scaffolds

Nanomechanical properties, such as nanohardness (H) and reduced Young’s modulus (Er) were performed using the nanoindentation technique (Oliver and Pharr methods) with a nanoindenter (NanoTest Vantage, Micro Materials, Wrexham, UK). A three-sided pyramidal diamond Berkovich’s indenter was used. Maximum force of indentation was 50 mN with 10 s of loading to maximum force time, 5 s dwell with maximum force, and 15 s of unloading time. The 15 independent measurements were performed with 20 µm distance between indentations. To calculate reduced Young’s modulus to Young’s modulus (E) Poisson’s ratio 0.3 was assumed.

### 2.5. Degradation of Fe Scaffolds

Degradation of the samples was studied by immersing cubic pieces of 6 mm × 6 mm × 6 mm in 10 mL 10 mM phosphate buffered saline (PBS) solution pH = 7.4 ± 0.02 in a sealed bottle at a temperature of 37 °C for 5 weeks. After each week, the samples were weighed on a microbalance. The corrosion rate was determined from the weight loss as follows:

(6) $$CR=\frac{m_{0}-m_{f}}{At}$$

where *CR* stands for the corrosion rate, *m*_0_ is the mass of sample before test, *m_f_* is the final mass after corrosion, *A* represents the surface area exposed to the PBS solution, and *t* is the immersion time.

### 2.6. Electrochemical Degradation of Fe Scaffolds

Electrochemical degradation was studied by potentiodynamic polarization (PDP) using a potentiostat (BioLogic SP-200). The study was carried out in a standard three-electrode system consisting of a working, a counter-electrode (platinum wire) and a reference electrode (chlorosilver electrode). The test was carried out in PBS solution at a constant temperature of 37 ± 1 °C. Before the measurement, the sample was immersed for 120 min to measure its open circuit potential (OCP). For the PDP test, the scan potential ranged from −250 mV to +250 mV relative to the stabilized OCP, measurements were conducted at a 0.167 mV·s^−1^ scan rate.

### 2.7. Albumin Adsorption

The protein adsorption studies were performed with ALB concentration in the range of 0.1–2.6 mg/mL. The mixtures were shaken at 120 rpm in a thermostated shaker for 2 h at 310 K. The protein concentration in obtained supernatants was measured using a Jasco V-750 UV-Vis spectrophotometer (Jasco Corporation, Tokyo, Japan) in a wavelength range of 200–450 nm (the area of the 280 nm band).

## 3. Results and Discussions

**Figure 2:**
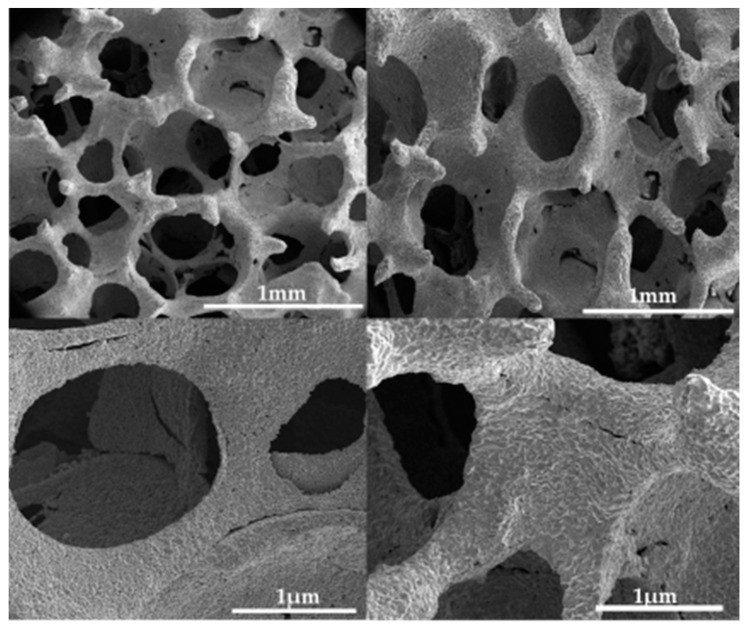
Scanning electron microscopy (SEM) images of synthesized Fe scaffolds.

The morphology of the fabricated iron scaffolds, resembling the structure of a sponge, is shown in Figure 2. As visible from the images, pore diameters are in the range of 245–360 µm. The size of macropores of the produced systems described in this paper, are very close to the pore sizes suggested for bone regeneration. Multiple studies have found that macroporous pores in the range of 150 to 360 µm are ideal [40]. This is motivated by the effect of the specific surface area, which is offset in larger pores by the increased potential for cell migration [7,40,41,42]. The oval-shaped pores with a diameter of 200 to 400 µm affect not only the function of osteoblasts but also chondrogenic differentiation [43]. Correlations between pore size and osteogenesis were observed only at the beginning of osteogenesis (up to 12 weeks), which proves the applicability of the biodegradable scaffold [44]. An appropriate shape that has a positive effect on osteogenesis and chondrogenesis is the first necessary condition for a resorbable bone implant.

**Figure 3:**
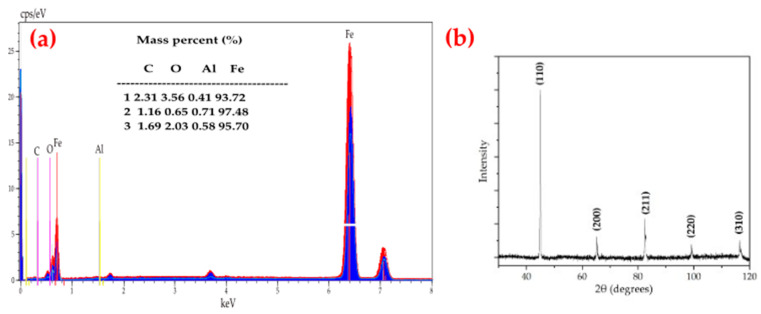
Energy dispersive spectroscopy (EDS) spectrum of synthesized Fe scaffolds (**a**) and their XRD diffraction characteristics (**b**).

Scaffolds produced by the method of a template immersed in the suspension of iron, result in the pore diameter of the scaffold formed being almost identical to the initial polyurethane template. In the used temperature program (Figure 1) in the scaffolds’ formation process, two stages can be distinguished: the first stage is the polymer burn-off temperature (500 °C) and the second one—the sintering temperature (1050 °C). To confirm the elemental purity of scaffolds, energy dispersive X-ray spectroscopy (EDS) was applied. Very intense lines in the EDS spectrum showed the presence of iron as a majority (Figure 3a). Moreover, the signals visible on the diffractogram (Figure 3) correspond to the pattern of pure iron. The intense (110) diffraction peak was obtained along with characteristic signals at (200), (211), (220), and (310) [45].

**Figure 4:**
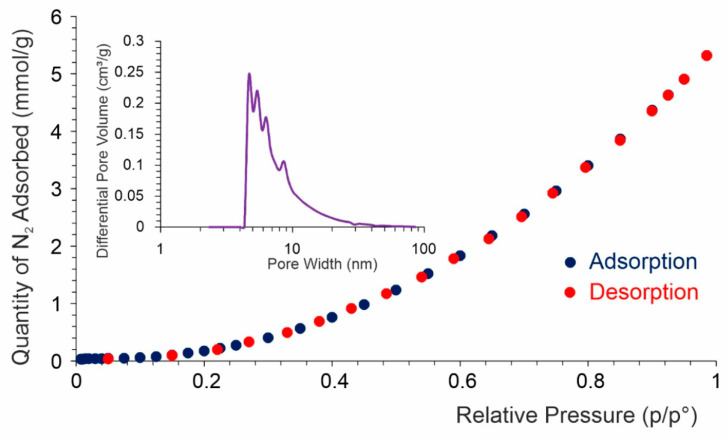
N_2_ adsorption-desorption isotherm (inset: pore size distribution).

Figure 4 shows the low temperature N_2_ adsorption isotherm. The amount of adsorbed N_2_ increases exponentially with pressure. Usually, this type of adsorption can be observed in non-porous or highly macroporous adsorbents. The lack of monolayer formation is very characteristic here, i.e., no “knee” in the isotherm curve. The overall mechanism is based on the fact that once the small droplet of adsorbate nucleates, further adsorption occurs more easily due to more adsorbate–adsorbate interactions than adsorbate–adsorbent.

Water contact angle measurements can only be performed on flat and stable surfaces. In our case, such analyzes could be difficult because of the sponge-like structure of the iron scaffold, as shown in Figure 2. The simplest solution is measuring the enthalpies of immersion in different probe liquids to characterize the surface energy components using the van Oss-Good-Chaudhury (VGC) approximation. The model easily separates the Lifshitz-Van der Waals component of the surface energy, $H_{S}^{LW}$, from polar terms, i.e., the acidic, $H_{S}^{+}$, and the basic, $H_{S}^{-}$, components, allowing calculation of the total surface energy, $H_{S}^{T}$ [46]. In addition, the separation of the acidic and basic components of the surface free energy, which is due to the ionic nature of the bonds present on the surface, provides information about the interactions of adsorbents with molecules on the surface [47]. The enthalpies of immersion in different probe liquids, namely, water, n-hexane, and formamide (*h_i_*), were measured and the results are shown in Table 1.

It is clear, from the results collected in Table 1, that Fe-foam is more of an acidic material than basic. The calculated $H_{S}^{+}$ is 33 times larger than $H_{S}^{-}$. Moreover, electrostatic interactions are dominate compared to non-polar ones. Its high water, work of adhesion proved good wettability of the material in a natural tissue environment and the calculated value of total surface energy is close to the literature data determined theoretically for the (1 0 0), (1 1 1), and (1 1 0) surfaces of iron [48] and calorimetric measurements [49].

The nanomechanical properties and energy calculation are presented in Table 2.

In this study, hardness values ten times higher than those of cortical bone were obtained. In Zysset et al. [50], hardness values ranging from 0.234 to 0.760 GPa were obtained, with Young’s modulus values similar for the values obtained for the tested materials (E value from 19.1 to 21.2 GPa). Thus, the results obtained are similar to those obtained with the same nanoindentation technique for a human femoral bone. The literature states that the Young’s modulus can range from 10–30 GPa for cortical bone [51]. For implants, it is crucial to obtain mechanical properties, especially Young’s modulus, as close as possible to the tissue in which the implant will be implanted, in this case—bone. It has been proven that significant differences in mechanical properties of an implant and bone provide a “shielding effect” and, consequently, may result in implant or bone damage [51,52,53]. Obtaining a Young’s modulus close to bone is due to the form of the material being tested. Manufacturing 3D scaffolds allows for lower Young’s modulus values compared to solid material (even 210 GPa for pure iron) [54,55].

Using the force-displacement diagram recorded during the measurement, it is possible to determine three types of energy. The area under the load curve indicates the total energy, the area under the unload curve indicates the elastic energy. The difference between the total energy and the elastic energy is the plastic energy. The H/E ratio (elasticity index) characterizes the ability of a material to resist elastic deformation [56]. This ratio can approximately characterize the wear resistance of a material. For tested materials, a H/E ratio value of 0.093 ± 0.027 was obtained. In Coy et al. for hard aluminum oxide-based coatings, H/E ratio values ranging from 0.03 to 0.08 were obtained. The H/E value for hard materials should be above 0.1, but values from 0.08 and above are reported in the literature as protective coatings with adequate wear resistance [57]. The high value in plastic/total energy ratio (ductility index, D) can be attributed to the proper fracture toughness. The ductility index for purely ductile materials is 1 and 0 is for purely elastic materials [58]. The D value obtained in this study may indicate a less ductile character of the material and a lower fracture toughness compared to the typical value for steel alloys (D > 0.9) [57]. On the other hand, the value of D is still above 0.5.

**Figure 5:**
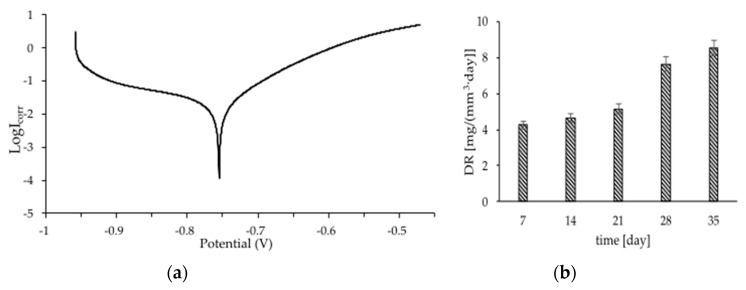
Potentiodynamic polarization curves (**a**) and the mass loss during immersion for 5 weeks (**b**) of the manufactured foams in PBS solution, pH 7.4 at 37 ± 1 °C.

Figure 5a shows the curve obtained by potentiodynamic polarization of the sample, which was previously stabilized for 120 min in PBS solution as a simulated body solution recorded at a scan rate of 0.167 mV/S. Table 3 shows the values of corrosion potential (ECORR) and corrosion current density (i_corr_), which were calculated from the intersection of anodic and cathodic Tafel lines extrapolation. The results of the immersion tests are shown in Figure 5b. A higher rate of loss in sample mass was observed every week. After the first week, punctuated color changes to brick-red were observed, after 3 weeks the samples had fully changed color. The highest weight loss was observed after 4 weeks, at this time the samples started to disintegrate. The corrosion rate results obtained for porous iron scaffold is higher than that reported for non-porous iron samples [35], this may be related to the increased surface area, macropores affecting the increased solvent access to the material and the sample roughness.

**Figure 6:**
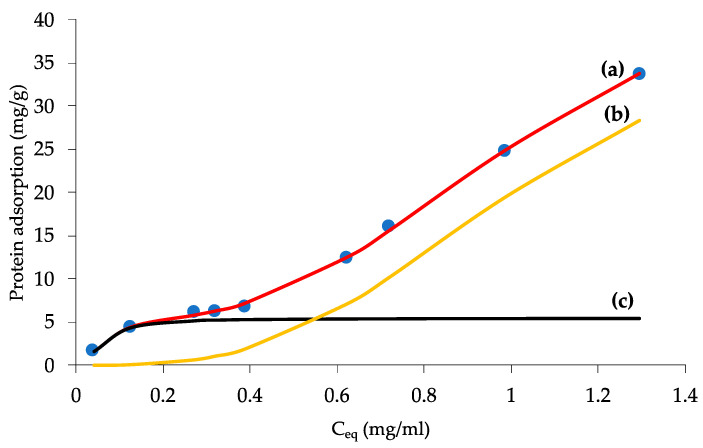
Albumin adsorption isotherms with fitted elements. (a) left-side and (b) right-side, of Equation (4); (c) fit of the experimental points (labelled as blue dots).

Good wettability of biomaterials is the prerequisite for further research. Nevertheless, it has been suggested that the nature of the surface allowing protein adsorption, beside the wettability itself, is important for cell attachment and later growth [34]. During osteogenesis cells never attach to a bare surface but to a surface previously covered with proteins adsorbed from biological fluids. This protein layer surely has a large influence on cell adhesion. When albumin and fibronectin are mixed, albumin adsorbs on hydrophobic surfaces and fibronectin on hydrophilic surfaces [47,59,60,61]. Taking into consideration resorbable abilities of the implant, we need to have a wettable surface where albumin is physically adsorbed. The results presented in Figure 6 prove that albumin is weakly adsorbed on the Fe-foam scaffold. This weak physical adsorption is successfully described by the bimodal Langmuir-Freundlich equation, which also correlates well with all the experimental data and results in Figure 6. The model was previously successfully harnessed for different adsorption data description, see e.g., [62]. Additionally, in previous studies [63,64], the model was used to quantify catalase and lysozyme adsorption on different carbonaceous materials with reasonable accuracy. The applied equation is represented by the expansion of the one proposed by Jeppu and Clement [65]; to bimodal form:

(7) $$Q_{eq}=\frac{Q_{m,1}{\left(K_{1}C_{eq}\right)}^{\frac{1}{n_{1}}}}{1+{\left(K_{1}C_{eq}\right)}^{\frac{1}{n_{1}}}}+\frac{Q_{m,2}{\left(K_{2}C_{eq}\right)}^{\frac{1}{n_{2}}}}{1+{\left(K_{2}C_{eq}\right)}^{\frac{1}{n_{2}}}}$$

where: *Q_eq_* is the amount of adsorbate adsorbed at equilibrium (mg_ALB_/g_Fe_); *Q_m_* is the maximum adsorbed capacity of the system (mg_ALB_/g_Fe_); *C_eq_* is the protein concentration in solution at equilibrium (g/L); *K* is the affinity constant between the adsorbate and the adsorbent (L/g); and *n* is the index of heterogeneity.

The fitted values of *Q_m,x_*, constant *K_x_* and the parameter *n_x_* (*x* = 1,2), are summarized in Table 4. A value of *n* = 1 suggests non-interacting sites, while 0 < *n* < 1 implies positive cooperation, while for *n* > 1, negative cooperation is expected during the adsorption process. The left part of Table 4 describes sites with higher Fe-foam surface to adsorbate affinity, typical for monolayer formation. On the contrary, lower *K*_2_ and *n*_2_ values shown on the right side define multilayer formation. It dominates in the higher *C_eq_* range. Interestingly, the values of *n_x_* appear here due to non-specific interaction, typical for physical adsorption. As a small surface area adsorbent, the Fe-foam allows ALB a high degree of freedom in movement, both rotational and lateral, which bodes well for future applications as a resorbable implant.

In a more complex, living, not-idealized system, adsorption on the surface of the substrate is performed primarily by proteins, whose concentration in solution is the highest. Next, the replacement of adsorbed proteins on the surface with those that have a higher affinity to the surface, occurs. In the literature, such a phenomenon is named the Vroman effect [66]. It has also been shown that for good osteogenesis, a synergistic effect between fibronectin and albumin during co-adsorption is also necessary; in effect, increasing cell adhesion and thus better and faster bone reconstruction [67,68,69,70]. The obtained results, i.e., physical adsorption of ALB on the Fe-foam, proved the potential applicability of the material as an effective implant. The full applicability will be checked in our further studies by the estimation of the rate of biocorrosion, the determination of the biocompatibility with cell lines, and by the verification of possible cytotoxicity.

## 4. Conclusions

The open-pore structure of pure iron scaffolds obtained by the repeated templating method appears to be a promising candidate as a biodegradable material for load-bearing implants in orthopedic applications. They showed adequate pore sizes, almost ideal for bone substitute material. They were characterized by having mechanical properties, especially Young’s modulus, close to the tissue in which the implant will be implanted, in this case—bone. It is important information, as using such a biomaterial can exclude the “shielding effect” and, consequently lower the chances of implant or bone damage. Moreover, a small surface area of Fe scaffold allows ALB a high degree of freedom in movement, both rotational and lateral, which bodes well for future applications as a resorbable implant. Nevertheless, further work is needed to manage the rate of biocorrosion, check biocompatibility with cell lines, and verify possible cytotoxicity.

## Figures and Tables

**Table 1:** The surface energy components, and the work of adhesion, W_adh_, of Fe-foam to water.

h_water_ [J/m^2^]	h_n-heptane_ [J/m^2^]	h_formamide_ [J/m^2^]	$\begin{array}{c} H_{S}^{LW}\\ \left[J/m^{2}\right] \end{array}$	$\begin{array}{c} H_{S}^{+}\\ \left[J/m^{2}\right] \end{array}$	$\begin{array}{c} H_{S}^{-}\\ \left[J/m^{2}\right] \end{array}$	$H_{S}^{T}\;$ [J/m^2^]	$\begin{array}{c} W_{adh}\\ \left[J/m^{2}\right] \end{array}$
−1.763 (0.21)	−0.352 (0.0201)	−1.951 (0.141)	0.827 (0.17)	10.67 (0.22)	0.278 (0.03)	2.550 (0.16)	1.881 (0.09)

**Table 2:** Nanomechanical properties and energy calculation results for Fe foam with standard deviation (*n* = 15).

Material	Fe Foams
Hardness [GPa]	2.584 ± 0.692
Young’s Modulus [GPa]	28.457 ± 5.601
E/H ratio [−]	0.093 ± 0.027
Plastic energy [nJ]	14.566 ± 3.448
Total energy [nJ]	25.095 ± 3.329
D ratio [−]	0.577 ± 0.085

**Table 3:** The avarage values for the corrosion potentials (Ecorr) and corrosion current resistance (i_corr_) obtained from the potentiodynamic polarization curves in PBS solution at 37.1 °C.

Material	E_CORR_ [mV]	i_corr_ [µA/cm^2^]
Fe foams	−755	68.6

**Table 4:** Fitted parameters of bimodal Langmuir–Freundlich equation (SD values are shown in parentheses) obtained for ALB adsorption on the Fe-foam.

*Q*_*m*,1_ [mg/g_Fe_]	*K*_1_ [L/g]	*n*_1_	*Q*_*m*,2_ [mg/g_Fe_]	*K*_2_ [L/g]	*n*_2_	R^2^
5.44 (0.11)	16.41 (1.77)	0.51 (0.02)	43.16 (0.81)	0.95 (0.05)	0.32 (0.02)	0.989

## Data Availability

Not applicable.
